# Integrating 4E cognition with science and technology studies: a framework for understanding AI applications

**DOI:** 10.3389/frai.2025.1545014

**Published:** 2025-07-21

**Authors:** Rasmus Gahrn-Andersen

**Affiliations:** Department of Culture and Language, University of Southern Denmark, Odense, Denmark

**Keywords:** performativity, 4E cognition, artificial intelligence, science and technology studies, integrative framework

## Abstract

The paper brings together two different theoretical strands of research, one from cognitive science, the other from Science and Technology Studies. The purpose in so doing is to uncover how cognition interrelates with socio-material practices and AI technology. An integrative framework is presented as a possible way for connecting the two strands while theorizing on their interrelations with AI.

## 1 Introduction

Artificial Intelligence is becoming increasing integral to human socio-material practices (Wellner, 2023). Whether such practices are instantiated as organizations, institutions or something else, it is evident that the spreading of AI is not restricted to isolated socio-practical domains (Peschl, 2024) and, moreover, that the ‘full potential’ of the technology yet has to be unleashed. In fact, the potentially unrestricted applicability of Large Language Models such as ChatGPT makes it imperative to recognize that AI technologies will end up being, not just used by humans as mere tools, but, even more so, come to interplay with the various ways in which our cognition and the practices we partake in evolve over time. The purpose of the present paper is to theorize on the interrelation of cognition, practices and AI technology, and to do so by engaging with two radical strands of research, one coming from cognitive science, the other from Science and Technology Studies (STS). The overall purpose^1^ is to bring these closer together and stress compatibility by presenting a framework for integrating some of their insights.

## 2 Theoretical background

### 2.1 4E cognition

Radical approaches to cognition in contemporary cognitive science are synonymous with the so-called 4E Cognition theories. Those in favor of a radical view generally assume that cognition is extended into the agent’s surroundings, embodied in that it involves not just the brain but also the body of the agent, enacted by the agent and, finally, embedded in actual contexts; rather than being simply reducible to an intracranial phenomenon (see, e.g., Marr, 1982). This contrasts with how those in favor of so-called Representational Theory of Mind (e.g., Fodor, 1981) consider cognition. They assume that “postulating representational (or ‘intentional’ or ‘semantic’) states is essential to the theory of cognition” (Fodor and Pylyshyn, 1988).

In pushing the 4E agenda, radical approaches differ from orthodox, representationalist approaches to cognition as they criticize the idea that cognition takes place strictly brain side and that it is bound to be based on representational content. As Cussins puts it, “A representational content is a presentation (or re-presentation) of the world in experience or in thought” (Cussins, 1992, p. 655). In this connection, there are different degrees of radicality amongst 4E approaches. Some proponents of anti-representationalism grant that so-called representation hungry problem domains do exist. The existence of these domains relates to the idea that those kinds of cognition that involve overtly reflective or deliberate attitudes such as planning or formulating propositions (cf. Clark and Toribio, 1994; Hutto and Myin, 2013) are bound to involve representations. Others, however, take the radical agenda a step further by seeking to eliminate representation hunger altogether (Degenaar and Myin, 2014; Kiverstein and Rietveld, 2018). Nevertheless, despite the fact that there is room for disagreements in relation to more ‘sophisticated’ kinds of cognition, what notions one should use for describing non-representational cognition (see, e.g., Hutto and Myin, 2013) etc. there is still a general, basic agreement amongst proponents of 4E that cognition can be theorized without presupposing the workings of mental content and representations (Gahrn-Andersen, 2021). However, what is currently missing from 4E approaches to cognition is that the interrelation between cognition and the unfolding of practices is generally not pursued in a systematic fashion. And this is despite the fact that it is recognized that the reliance on affordance relations through engagements with tools, things, computers etc. is not only characteristic of a situation, but trans-situational in the sense of being tied up with practices which are irreducible to the particularities of a situation. As van Dijk and Kiverstein put it, affordances “are situated, but concretely available aspects of the sociomaterial environment to coordinate to. We can see that they function in these manifold ways if we zoom out on our practices in space and time to notice how chairs, doors, benches, paths and ponds are entangled within and across concrete situations” (van Dijk and Rietveld, 2017, p. 6). Despite not having engaged with the issue of trans-situationality of cognition from a constitutive point of view (one could perhaps claim that Hutchins does so but descriptively), 4E theories provide good insights into mechanisms that allow cognition to unfold as a situated, practical phenomenon.

#### 2.1.1 Know-how and affordances

Know-how is important in the sense that it enables flexible adaptive behavior; both in socially saturated situations and beyond. Such knowhow lends itself to embodied expectations (which are often tacit and not previously articulated) and possibilities for actions, and it characterizes even our plain, everyday enacted perceiving (Hutto, 2005, p. 391). Indeed, such intentional directedness is viewed as relying on know-how or being skilled in that it entails “coordinating with multiple affordances simultaneously in a concrete situation” (van Dijk and Rietveld, 2017, p. 8). Affordances, on this view, are opportunities for actions that exist as a relation between an agent and its environment (Gibson, 1979). In other words, cognition relies both on agent-specific skills and capacities as well as features in the material world; the world thus come to play a constitutive role to cognition.

#### 2.1.2 Practical understanding

In building on key insights from the early Heidegger, Noë (2015) makes the claim that our perception and practical dealings more generally are guided by our practical understanding. Noë argues: “What makes [understanding] practical […] is that it is the gearing in or putting to work of one’s understanding in the absence of any call for, or even space for, reflection or judgement” (2015, p. 3). In other words, it happens in the ‘absence of deliberation’ (2015, p. 4) but nevertheless allows us to discriminate practically relevant affordances from irrelevant ones. Moreover, it allows for something to afford X in a given situation and not Y or Z. Indeed, practical understanding can be seen as the enabler of know-how in that it allows know-how to be used ‘correctly’ and, thus, for agents to tacitly adopt their skills through engagements with affordances, not just to what the situation affords, but also, to what makes affordances practically entangled with other situations relative to the practice in question (see, Hutchins, 1995 for numerous examples of such understandings at play in a tightly coupled organization or Heidegger, 2010 for a more technical argument on how things stand in practical relevance to other things).

### 2.2 Performativist STS

Just as in the case of cognitive science, contemporary Science and Technology Studies (STS) is characterized by an internal, paradigmatic shift. Indeed, some proponents of radical cognition have been stressing a degree of compatibility (De Jesus, 2018). Also, in this case, the split consists between, on the one hand, those who argue in favor representational approaches to the study of scientific practices and technology-use more generally and, on the other, those advocating in favor of a performative approach. The representational idiom is characterized by a detached view of technology use, where the human agent is seen as separate from the world. This detachment entails that the agent is able to reason or cognize about worldly affairs in an objective manner; basically observing states of affairs from a distance (Jensen, 2004) Performativists such as Haraway (1991), Pickering (1995), Latour (1999) and many others, on the other hand, emphasize the importance of practical ontologies. Such ontologies are considered as alternatives to certainties of knowledge (i.e., epistemology) and mental representations. Moreover, proponents of performativism argue that we should not impose an analytical distinction between social facts and cognitive facts since the two are enmeshed in the outset (cf. Jensen, 2004, p. 237). In terms of thematizing the trans-situationality of practices, we find at least two useful notions; not only for exploring practical activity as something that unfolds over time, but also useful notions bringing 4E insights together with performativist ones.

#### 2.2.1 Activity trails

Based on the works of Cussins (1992), activity trails are minimally defined as patterns or ‘forms of guidance’ that move through environments of activity. They not only “lend stability” to categories such as ‘the political’, ‘the technological’ and ‘the social’ (Jensen, 2017, p. 631) but also help to escape the traps of humanist approaches. Simply, they do not take the human agent as their analytical ground. Consequently, they enable “decentred analyses in which subject and object formations are emergent outcomes of material—relational processes” (Jensen, 2017, p. 631). They are also used to counter claims that performativist positions entail “radical relativism” by embracing practical ontologies instead of objectifying epistemologies (Gad et al., 2015). Indeed, Jensen appeals to activity trails in infrastructure configurations, showing that “they help recreate not only the ‘objective’ space of the city, but also the ‘subject positions’ of those who depend on these infrastructural arrangements” (Jensen and Morita, 2017, p. 8). Activity trails connect what is objective and subjective, and they go beyond focusing only on the thinking person as the center of actions and events.

#### 2.2.2 Conjunctions

‘Conjunctural events’ or simply ‘conjunctions’ describe how novelties come about “out of various contingent combinations of heterogeneous instruments, temporal and spatial dispositifs, procedures and techniques; something else than what caused it, which has its own specific historical trajectory; […] something which does not bring together already existing objects, subjects and social groupings” (Hennion and Grenier, 2000, p. 9). But the relevance of conjunctions goes beyond artistic practices to basically involve any occurrence where different socio-material entanglements generate novelties including, for instance, as solutions to deltas in crisis such as the Mekong and Chao Phraya (Jensen and Morita, 2020).

## 3 Theoretical integration

**Figure 1:**
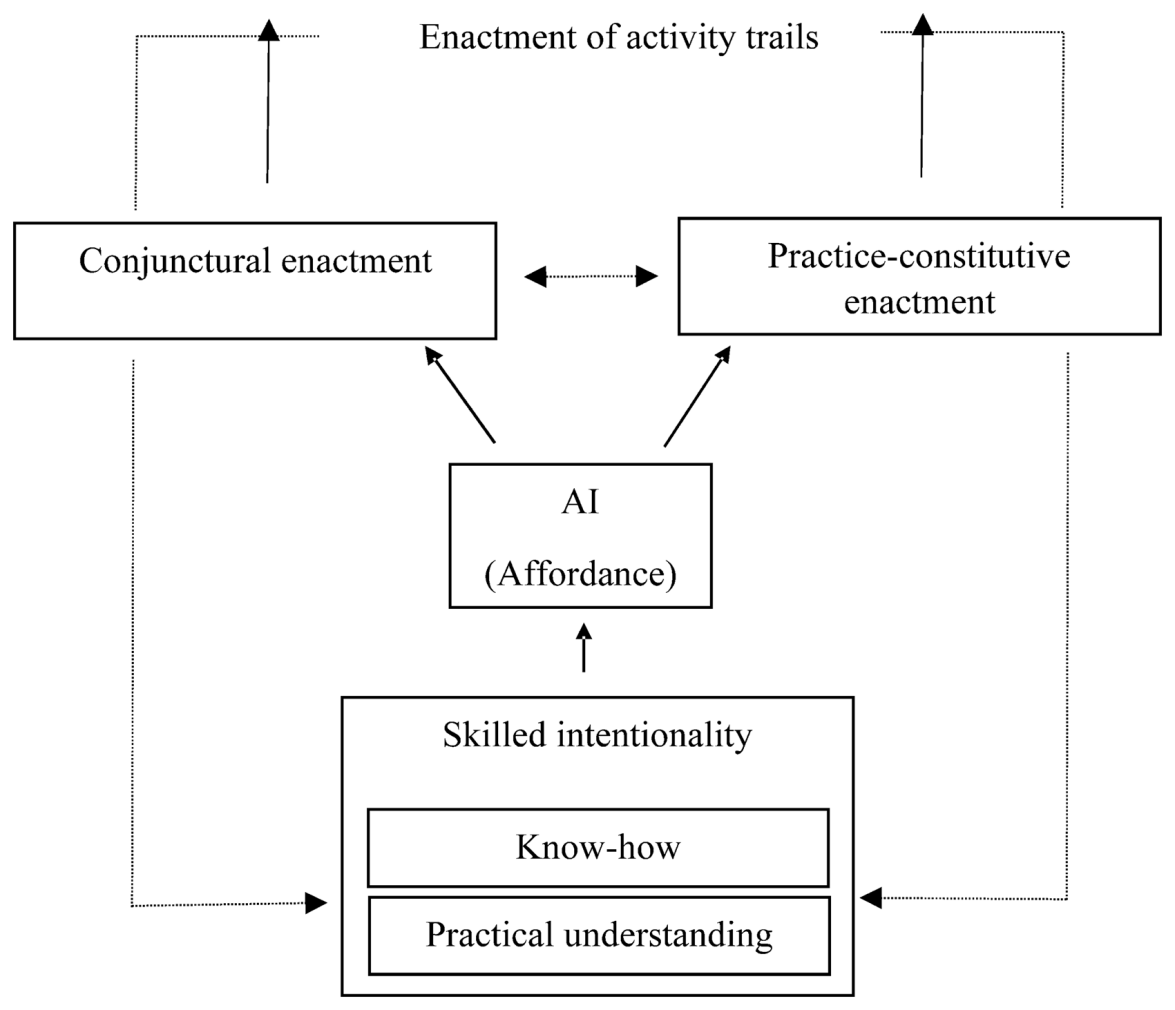
The framework illustrates how engagements with AI are shaped by both agent-relative dispositions and broader social practices. On the agent side, we observe skilled intentionality, which enables meaningful interaction with AI through a twofold structure of know-how and understanding. Although this structure is agent-relative, it is neither inert nor static: agents adapt to the real-world effects generated by their use of AI technologies (as represented by the two downward-pointing arrows). In terms of what AI affords, we can conceptualize the agent’s enactments in two ways: as conjunctive or practice-constitutive. Conjunctive enactments involve the generation of novelty—such as in leisure or artistic expression—without directly feeding into existing practices. In contrast, practice-constitutive enactments are integrated into established practices or may even contribute to the emergence of new practices. In any case, the outcomes of human-AI interactions give rise to real-world effects through activity trails.

AI technologies are world-involving (Peschl, 2024) in the sense that we make their outcomes influence or be influenced by the unfolding of everyday practices. AI can support or replace actions, serving as affordances to a practice. For instance, one can ask ChatGPT to generate a recipe, draft a program for a conference or write a speech. Based on the output generated by the AI, the human user can decide to enact aspects hereof, not just onto their surroundings, but also in manners whereby they tie in with particular features of an existing practice (Sadek et al., 2024). The technology enables users to offload cognitive effort (Chemero, 2009) by speeding up tasks, improving performance and planning, making some tasks unnecessary, and even creating new ones. In short, they allow for different relationalities between agents and aspects of their environments (Figure 1).

We engage with AI technologies in ways that reflect our skilled intentionality. Consider the recent trend of people using generative AI to create action figures of themselves or others. This phenomenon may have originated accidentally—perhaps someone was casually experimenting and found the results intriguing enough to share on social media. Countless other artistic creations fail to achieve similar viral impact. Regardless, this trend can be used to showcase the intentionality that underlies creative engagements with LLM-based technologies. Such intentionality is rooted in a twofold structure: know-how and practical understanding. In the case of the action figures, it involves not only familiarity with prompt engineering—the skill of crafting effective prompts—but also a practical grasp of what qualifies as an ‘action figure’. Furthermore, it is the same sort of practical understanding that allows us to critically evaluate AI outputs (for a concrete example, see Gahrn-Andersen, 2025).

When part of a broader trend, the users employ their expertise not only in operating the AI system but also in integrating its outputs meaningfully into real or virtual contexts. Generative models like ChatGPT or DALL·E 3 can be used for artistic expression or simply to pass the time. This highlights the fact that their outputs do not necessarily have immediate practical utility. Instead, they may be artistic, exploratory, or idiosyncratic in nature. AI technologies can thus be used conjuncturally—creatively or in ways that are not directly tied to existing practical configurations. While these uses may not feed into established practices, except perhaps those related to art (cf. Hennion and Grenier, 2000; Chatterjee, 2022), they still hold potential significance. Conjunctural uses can serve as sites of innovation, bringing novelty to established practices.

Although the creation of an action figure using AI need not afford immediate practical use, the recent trend shows that it can serve different purposes—such as self-promotion on platforms like Instagram or Facebook, or in more professional contexts: on LinkedIn. The popularity of creating self-crafted action figures as a ‘social media trend’^2^ shows how this phenomenon interrelates with posting practices on different social media platforms. This reflects the trans-situationality of the practice: not only is it re-enacted across different platforms, but it also persists and evolves across these different contexts and over time. While the results are partially determined by how the AI functions internally, their perceived quality and relevance also depend on the user’s platform-specific knowledge and disposition—how and where the generated content is shared. Finally, it’s important to note that AI use evolves so rapidly that ostensive descriptions—explicit, shareable instructions—for how to prompt LLMs to generate specific outputs, such as action figures, are now readily available.^3^ The know-how required to generate an action figure of a particular person has shifted from being predominantly idiosyncratic and relative to the actions of particular individuals to becoming general. Prompt engineering has matured into a recognizable practice, complete with guidelines and informed tips. As a result, the ‘practice-constitutive enactment’ of generating such content has been explicated and made ostensible, making it replicable and teachable. However, if one does not want to bother with fine tuning prompts for ChatGPT or DALL·E 3, one can instead use Flux AI’s Action Figure Generator which is designed specifically for the purpose of generating action figures. Here, one simply provides basic information about the kind of figure one intends to create a representation of, without having to tell the AI what constitutes an action figure, from what angle etc. Thus, one does not need to have refined prompting skills in order to make an action figure.

Considering how Flux AI promotes its services, it becomes clear that AI-generated material, such as images of custom-made action figures, can support a wide range of practical applications and potentially catalyze diverse forms of engagement, constituting different activity trails. For example, their website highlights different practical domains such as content creation by influencers, gift giving, collecting, and use by small businesses to design mockups for custom merchandise or promotional collectibles.^4^ In general terms, the agent’s capacities and dispositions constitutes what an AI system may offer in a given context, lending support to decision-making (Eisbach et al., 2023) and triggering new paths of action. These new activity trails not only shape how the practice evolves over time but also influence the user’s own skills and understanding, allowing these to grow as well (Benvenuti et al., 2023).

## 4 Conclusion

The paper shows that theoretical notions from 4E Cognition theories and performativist STS can be brought together in a fruitful interplay for the purpose of exploring how cognition and practices interrelate in the use of AI. The proposed framework can inspire future research in both camps. Specifically, it can inspire proponents of 4E cognition theories to extend their concepts into the realm of AI and human socio-material practices more general. For proponents of performativist STS, on the other hand, it effectively brings the cognitive back into the loop by demonstrating that cognitive phenomena can be treated in non-representationalist (or, simply: performativist) terms and that cognitive mechanisms such as know-how and practical understandings relate to AI-use in the enactment of practices.

## Data Availability

The original contributions presented in the study are included in the article/supplementary material, further inquiries can be directed to the corresponding author/s.

## Footnotes

^1^This paper aim is to offer conceptual synthesis and critical reflection, and not an empirical analysis. Accordingly, it does not include formal methodological details such as inclusion/exclusion criteria, search strategies, or coding protocols. The mapping of 4E cognition concepts onto STS constructs and vice versa is interpretive and grounded in my own critical engagement with the literature.

^2^https://www.wired.com/story/chatgpt-image-generator-action-figure-privacy/ [Accessed June 10, 2025].

^3^https://www.forbes.com/sites/lesliekatz/2025/04/15/ai-action-figure-trend-explained---and-how-to-make-your-own/ [Accessed June 10, 2025].

^4^https://fluxai.art/features/ai-action-figure-generator [Accessed June 10, 2025].
